# Editorial: Multimodality therapy for older cancer patients

**DOI:** 10.3389/fonc.2024.1487783

**Published:** 2024-09-30

**Authors:** Nam P. Nguyen, Mohammad Mohammadianpanah, Meritxell Arenas, Vincent Vinh-Hung

**Affiliations:** ^1^ Department of Radiation Oncology, Howard University, Washington, DC, United States; ^2^ Colorectal Research Center, Department of Radiation Oncology, Namazi Hospital, Shiraz University of Medical Sciences, Shiraz, Iran; ^3^ Department of Radiation Oncology, Sant Joan de Reus University, University of Rovira, I Virgili, Tarragona, Spain; ^4^ Department of Radiation Oncology, Centre Hospitalier Public du Contentin, Cherbourg-en-Contentin, France

**Keywords:** older, immunotherapy, radiotherapy, synergy, multimodality

The management of locally advanced cancer frequently requires a multimodality approach due to high rates of loco-regional recurrences and/or distant metastases (1). Surgery, followed by postoperative radiation, concurrent chemotherapy and radiotherapy, and preoperative chemoradiation are standard approaches for these patients, depending on the anatomic site. However, older cancer patients with locally advanced disease are often not ideal candidates for surgical resection due to pre-existing comorbidities, a high risk of postoperative complications, and poor survival rates after treatment (2). Additionally, frail patients may not benefit from chemotherapy due to a high mortality rate and frequent hospitalizations during treatment (3). As a result, they are often denied curative treatment as clinicians are concerned about their ability to tolerate it.

Innovative therapies such as immunotherapy with immune checkpoint inhibitors (ICIs) may offer a curative option with minimal morbidity when combined with new radiotherapy techniques like image-guided radiotherapy. Immunotherapy is well -tolerated and has been reported to be effective for older cancer patients, comparable to its effectiveness in younger patients (4). It is most effective among patients with positive program death ligand 1 (PD-L1) expression, defined as 1% or above. However, patients who lack PD-L1 in their tumors may still benefit from immunotherapy if they receive radiotherapy first. Preclinical and preliminary clinical data suggest that radiotherapy may increase PD-L1 expression in tumors, as cancer cells produce an immune -suppressive environment to escape destruction by CD-8 T cells (5).

The best illustration of the synergy between radiotherapy and immunotherapy is reflected in the model of renal cell carcinoma, which is reported to be radio-resistant. This resistance often requires a high dose of radiation, which can potentially damage surrounding normal organs such as the liver and the small intestine. Additionally, there is a high rate of distant metastases in tumors with high risk features such as large size and poorly differentiated histology. Historically, patients who develop distant metastases had a very poor outcome due to the tumor resistance to chemotherapy. The survival of those patients has significantly improved with ICIs. The combination of ICIs and radiotherapy is also very well tolerated and effective for patients with distant metastases. Thus, at least in theory, immunotherapy and modern radiotherapy techniques such as stereotactic body radiotherapy (SBRT), which delivers a high curative dose of radiation with minimal toxicity, should improve local control and survival for renal cancer patients with locally advanced disease (Nguyen et al.). In another example, the combination of ICIs with radiotherapy for locally advanced bladder cancer has produced an 81% biopsy proven complete response (CR), which is significantly higher than the responses reported after concurrent chemoradiation or neoadjuvant immunotherapy (Nguyen et al.). For selected patients with locally advanced rectal cancer, immunotherapy alone or combined with chemotherapy and radiotherapy may lead to organ preservation in a disease that traditionally require surgery for local control (6).

Therefore, the judicious sequencing of immunotherapy and radiotherapy may benefit most patients with locally advanced cancers, regardless of their PD-L1 status. Specific protocols need to be developed for each tumor type for older cancer patients, taking into account their frailty status to avoid unnecessary treatment toxicity (7). As an international organization dedicated to the care of older cancer patients, the International Geriatric Radiotherapy Group (http://www.igrg.org) is committed to conducting prospective trials combining radiotherapy and immunotherapy for this vulnerable population (8). The data obtained may allow us to optimize treatment strategies for older cancer patients, improving outcomes and quality of life for these individuals.
